# A de novo LDLR mutation in severe familial hypercholesterolemia: case report, functional characterization, and a personalized gene correction strategy exploration

**DOI:** 10.3389/fcvm.2026.1814798

**Published:** 2026-06-09

**Authors:** Shuran Zhang, Wenming Huang, Haoqiang Chen, Ninghui Mu, Le Chang, Baosheng Zhu, Jinman Zhang, Ying Chan

**Affiliations:** 1 Medical Genetics Department of The First People's Hospital of Yunnan Province/Affiliated Hospital of Kunming University of Science and Technology, Key Laboratory Reproductive Health and Birth Defects Prevention and Control in Western China, National Health Commission, Key Laboratory of Birth Defects and Genetic Diseases of Yan Province, Kunming, Yunnan, China; 2 School of Medicinie, Kunming University of Science and Technology, Kunming, Yunnan, China

**Keywords:** familial hypercholesterolemia, gene correction, low-density lipoprotein receptor, pathogenicity validation, prime editing

## Abstract

**Background:**

Familial hypercholesterolemia (FH) is a genetic disorder of lipid metabolism characterized by elevated plasma low-density lipoprotein resulting in cardiovascular disease (CVD). The harmful mutations of LDLR are the main cause of FH. Especially, there is no effective treatment options for homozygous FH (HoFH) patients. Numerous FH cases have been reported, but most mutations remain unvalidated and lack gene correction studies. The study aims to assess the pathogenicity of a novel mutation, *LDLR* c.331C>T (p.Gln111Ter), and seek its gene correction strategy.

**Methods:**

The study systematically evaluated a female HoFH patient and her family. Using CRISPR/Cas9 technology, a Huh7 cell line carrying the point mutation was constructed. The impact of this mutation on LDLR protein expression was confirmed by qPCR, Western blot (WB), and immunofluorescence. A high-fidelity gene correction system targeting the *LDLR* c.331C>T point mutation was established based on the prime editing (PE) technology.

**Results:**

The HoFH patient exhibited a biallelic *LDLR* mutation comprising an *LDLR* c.1693_1696 del GGCA inherited from her mather and a *de novo LDLR* c.331C>T (p.Gln111Ter) mutation. *In vitro* validation indicated that the mutation impaired normal LDLR protein expression, and the candidate gene editing system achieved approximately 98% correction efficiency.

**Conclusion:**

*LDLR* c.331C>T is a likely pathogenic mutation, which canbe precisely corrected by PE technology. The study expands the spectrum of likely pathogenic mutations in FH and holds promise for personalized, precise gene therapy through customized therapeutic systems, potentially alleviating or curing HoFH—a current challenge in conventional clinical management.

## 1 Introduction

Familial Hypercholesterolemia (FH) is an autosomal dominant disorder of lipid metabolism characterized by markedly elevated plasma low-density lipoprotein cholesterol (LDL-C), pathognomonic xanthomas, and premature atherosclerotic cardiovascular disease (ASCVD) (1). FH represents a significant burden on global public health systems. Based on inheritance patterns, FH is primarily categorized into heterozygous FH (HeFH) and homozygous FH (HoFH). HeFH results from a pathogenic mutation in a single allele, whereas HoFH arises either from biallelic pathogenic variants in the same gene (true homozygote) or from two distinct pathogenic variants in alleles of the same FH-associated gene (compound heterozygote) (2, 3). The prevalence of HoFH ranges from approximately 1/160,000–1/1,000,000, which is considerably lower than that of HeFH (1/200–1/500) (4, 5). Clinically, HoFH manifests with more severe phenotypes and currently lacks consistently effective therapeutic options. The identification, functional validation, and targeted correction of *LDLR* mutation sites are therefore crucial for advancing clinical diagnosis and therapeutic development in FH.

FH is primarily caused by mutations in genes involved in low-density lipoprotein cholesterol (LDL-C) metabolism, including those encoding the low-density lipoprotein receptor (*LDLR*), apolipoprotein B (*ApoB*), proprotein convertase subtilisin/kexin type 9 (*PCSK9*) (6, 7), and LDL receptor adaptor protein 1 (*LDLRAP1*) (8–10). Among these, *LDLR* mutations account for approximately 90% of FH cases (11). The *LDLR* is located on chromosome 19p13.1–p13.3, spans approximately 45 kb, and comprises a ligand-binding domain (containing seven LDL-A repeats) (12), an EGF precursor homology domain, an O-glycosylation domain, a transmembrane domain, and a cytoplasmic domain (13, 14). Each domain serves a distinct function, and alterations in any domain may impair LDLR activity. Pathogenic *LDLR* mutations can disrupt function at any stage of the LDLR cycle, affecting the LDL uptake system and leading to inefficient clearance of LDL-C (15). Consequently, elevated plasma cholesterol levels contribute to the development of atherosclerotic cardiovascular diseases (16).

Although thousands of *LDLR* mutation sites have been reported globally (17), the continued discovery of novel variants and their functional validation remains crucial for refining the genetic spectrum of FH and advancing precision diagnostics and treatment. The often-subtle early symptoms of FH contribute to significant diagnostic challenges, with over 90% of cases remaining undiagnosed. Early diagnosis of FH is of great significance for preventing morbidity and mortality (18). Therefore, improving the diagnostic rate is a key priority in clinical management (19). The ongoing identification and timely functional characterization of novel mutations can enhance diagnostic accuracy.

This is particularly relevant for patients with homozygous FH (HoFH), who typically present with severe clinical phenotypes and show poor response to standard lipid-lowering therapies, such as high-intensity statins and PCSK9 inhibitors (20). Elucidating the pathogenic mechanisms of novel mutations at the molecular level and developing precise correction strategies for these point mutations not only provide definitive genetic diagnostic evidence for patients and their families but also establish an essential theoretical and technical foundation for exploring novel individualized gene therapy interventions.

Accordingly, the study presents a case of HoFH harboring a novel nonsense mutation in *LDLR* (c.331C>T, p.Gln111Ter). Functional validation was conducted in a hepatocyte-derived model system carrying this point mutation, followed by the development of a targeted gene correction strategy based on PE. Our work aims to enhance the clinical diagnosis of FH and to propose a potential personalized gene-based therapeutic approach for HoFH.

### 1.1 Case presentation

#### 1.1.1 Patient information and disease phenotype

**Figure 1:**
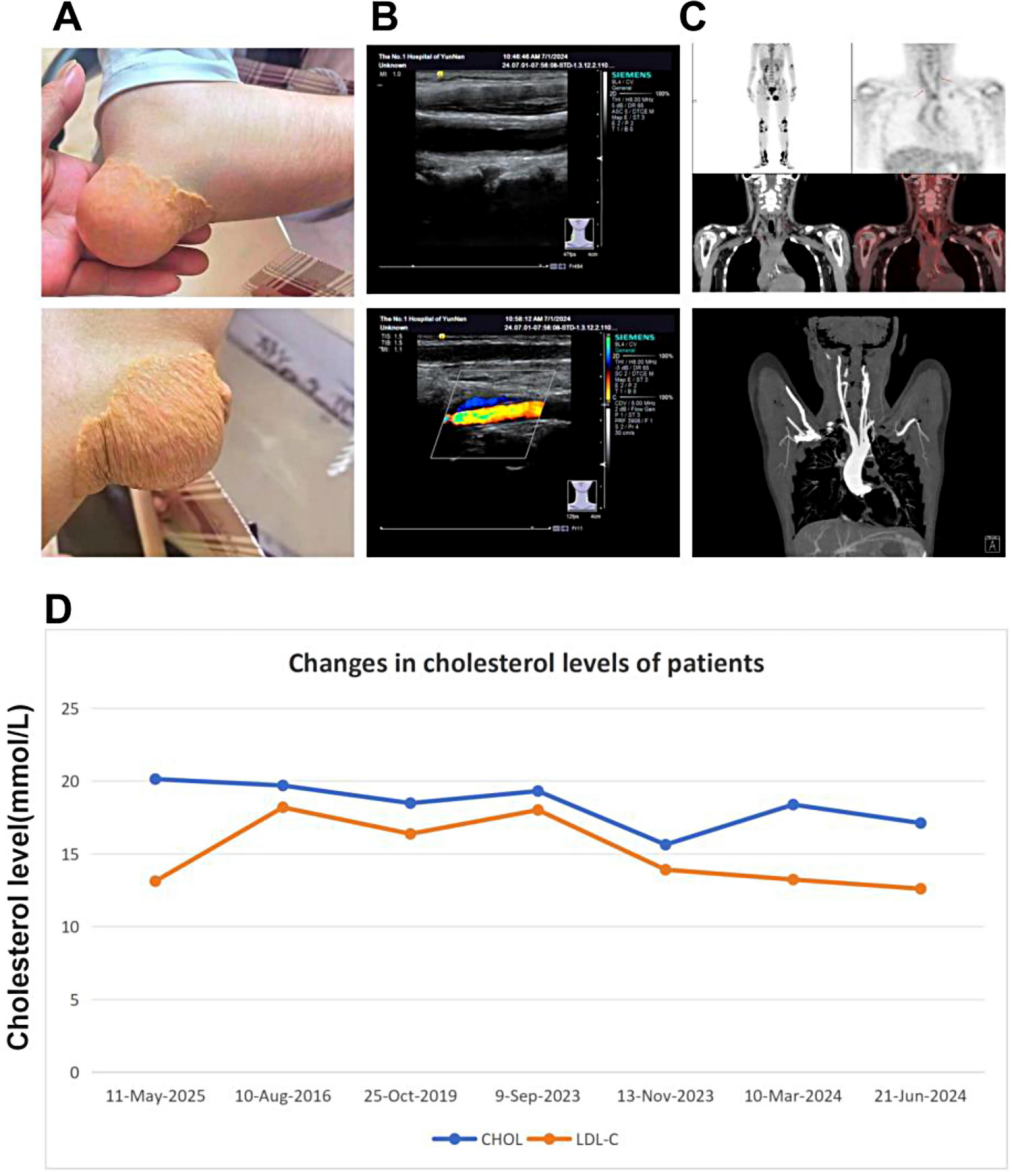
Biochemical and imaging findings of the patient. **(A)** Xanthomas present bilaterally at the elbows. **(B)** B-mode ultrasound of systemic vasculature shows bilateral carotid atherosclerosis. **(C)** PET-CT images demonstrate segmental wall thickening and calcification of the aorta, common carotid arteries, internal carotid arteries, and iliac arteries, consistent with atherosclerotic plaques. **(D)** Longitudinal profile of total cholesterol (CHOL) and low-density lipoprotein cholesterol (LDL-C) levels over more than ten years.

The patient in the study is a 17-year-old female borned in 2008 and diagnosed as HoFH at the Department of Medical Genetics, The First People's Hospital of Yunnan Province in 2018. At the age of 3, she developed hypertension and hypercholesterolemia, and smooth masses were noted bilaterally at the elbows (Figure 1A). In 2014, a pathological examination of a dorsal foot skin biopsy performed at affiliated Xinhua Hospital,School of Medicine in Shanghai Jiao Tong University, which indicated juvenile xanthogranuloma, consistent with xanthoma. In 2015, she was admitted to Kunming Children's Hospital due to recurrent headaches, vomiting over a half-month period, and three episodes of convulsions. She was diagnosed as hypertensive encephalopathy, acute glomerulonephritis, multiple lipomas, hyperlipidemia, and streptococcal infection, and was discharged after 6 days of hospitalization with improved symptoms.

In January 2016, she visited Beijing Friendship Hospital, Capital Medical University. Blood biochemistry revealed that total cholesterol (CHOL) was 19.76 mmol/L and LDL-C is 13.02 mmol/L (normal reference range: 1.27–3.12 mmol/L) (Figure 1D). Systemic vascular ultrasound indicated bilateral carotid atherosclerosis. Given the diagnosis of FH-related lipid metabolism disorder, liver transplantation was recommended to improve her lipid profile; however, the parents declined due to her young age. After discharge, she was regularly treated with simvastatin and ezetimibe for lipid management, with periodic blood tests showing persistently poor cholesterol control. Evolocumab was later added, resulting in a modest reduction in LDL-C, though levels remained well above normal.

In 2018, genetic testing confirmed a diagnosis of HoFH caused by compound heterozygous mutations in *LDLR*, indicating a very high risk of cardiovascular disease. For further evaluation, she was admitted to the cardiology department in June 2024 for biochemical and imaging assessments of her lipid levels and vascular damage. CT scans showed multiple calcified plaques in the aortic root, aortic arch, and descending aorta. The segmental wall thickening and calcification in the aorta, common carotid arteries, internal carotid arteries, and iliac arteries were revealed after PET-CT test, suggestive of atherosclerotic plaques (Figure 1C). An increased blood flow velocity in the descending aorta (suggesting possible stenosis) was indicated through Echocardiography, while coronary imaging results showed no significant abnormalities. Multiple bilateral carotid plaques were demonstrated by Ultrasound scan (Figure 1B).

Over the past 12 years, the masses behind both elbows have continued to grow, and smooth nodules have successively appeared in the knee joints, ankle joints, metacarpophalangeal joints, proximal interphalangeal joints, and distal interphalangeal joints. The CT, ultrasound, biochemical, and imaging findings collectively reflect a disease phenotype consistent with severe hyperlipidemia.

### 1.2 Analysis of clinical data

#### 1.2.1 Genetic testing results

**Figure 2:**
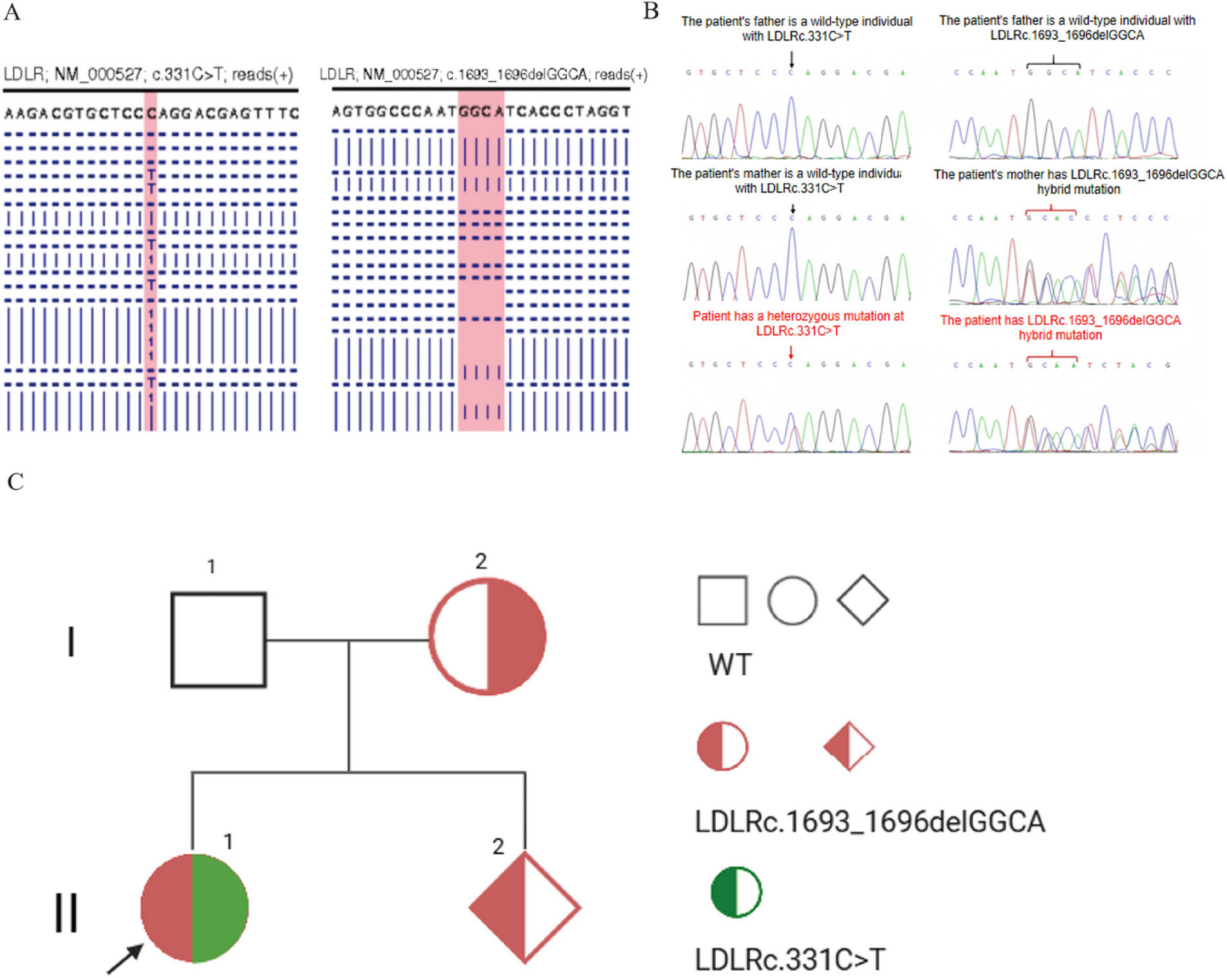
Family diagram. **(A)** Information on two *LDLR* mutation sites. **(B)** Sanger peak diagram of *LDLR* locus sequencing in the patient's family. **(C)** Family pedigree of the patient.

In December 2018, genetic sequencing of FH-related genes (*APOB*, *LDLR*, *LDLRAP1*, *PCSK9*) was performed, followed by whole-exome and familial variant segregation analysis. The results indicated no abnormalities in the father. A frameshift mutation in exon 11 of *LDLR* c.1693_1696delGGCA (p.Gly565SerfsTer3, heterozygous) was identified in the mother and the fetus, with no previously reported pathogenicity for this variant. In addition to inheriting the maternal frameshift mutation, the proband was found to carry a de novo nonsense mutation in exon 4 of *LDLR*: c.331C>T (p.Gln111Ter, heterozygous) (GRCh38: chr19:11215913) (Figure 2). This mutation is predicted to introduce a premature termination codon, resulting in a truncated LDLR protein that may significantly impair its structure and function.

Lipid profiling of the family showed elevated total cholesterol levels in the mother, maternal grandfather, and maternal uncle; However, except for the patient's mother, none underwent genetic testing. Without accounting for age-related changes, the origin of the maternal the frameshift mutation and the precise cause of familial hypercholesterolemia remain undetermined. Nor can we completely exclude the possibility of other, as yet unidentified, hypercholesterolemia-associated genetic variants (e.g., mutations in APOB or PCSK9) existing in this family. Consequently, the precise contribution of the frameshift mutation alone to the observed hypercholesterolemia in affected family members cannot be definitively established from the current pedigree data alone. Notably, the proband's mother, who carries only the frameshift mutation (*LDLR* c.1693_1696delGGCA), exhibited a mild clinical phenotype that was well-controlled with medication. This observation strongly suggests that the frameshift mutation alone is insufficient to drive the severe HoFH phenotype. Consequently, the *de novo* nonsense mutation (*LDLR* c.331C>T) is likely the primary pathogenic driver of the patient's early-onset and severe clinical presentation. Nevertheless, the present study cannot completely exclude the possibility of a synergistic pathogenic effect between the two mutations in the patient. Independent functional validation of the frameshift mutation will be critical to clarify this issue and will provide the necessary basis for a comprehensive understanding of the pathogenic mechanisms underlying this compound heterozygous mutation.

#### 1.2.2 Functional implications of the *de novo* mutation

**Figure 3:**
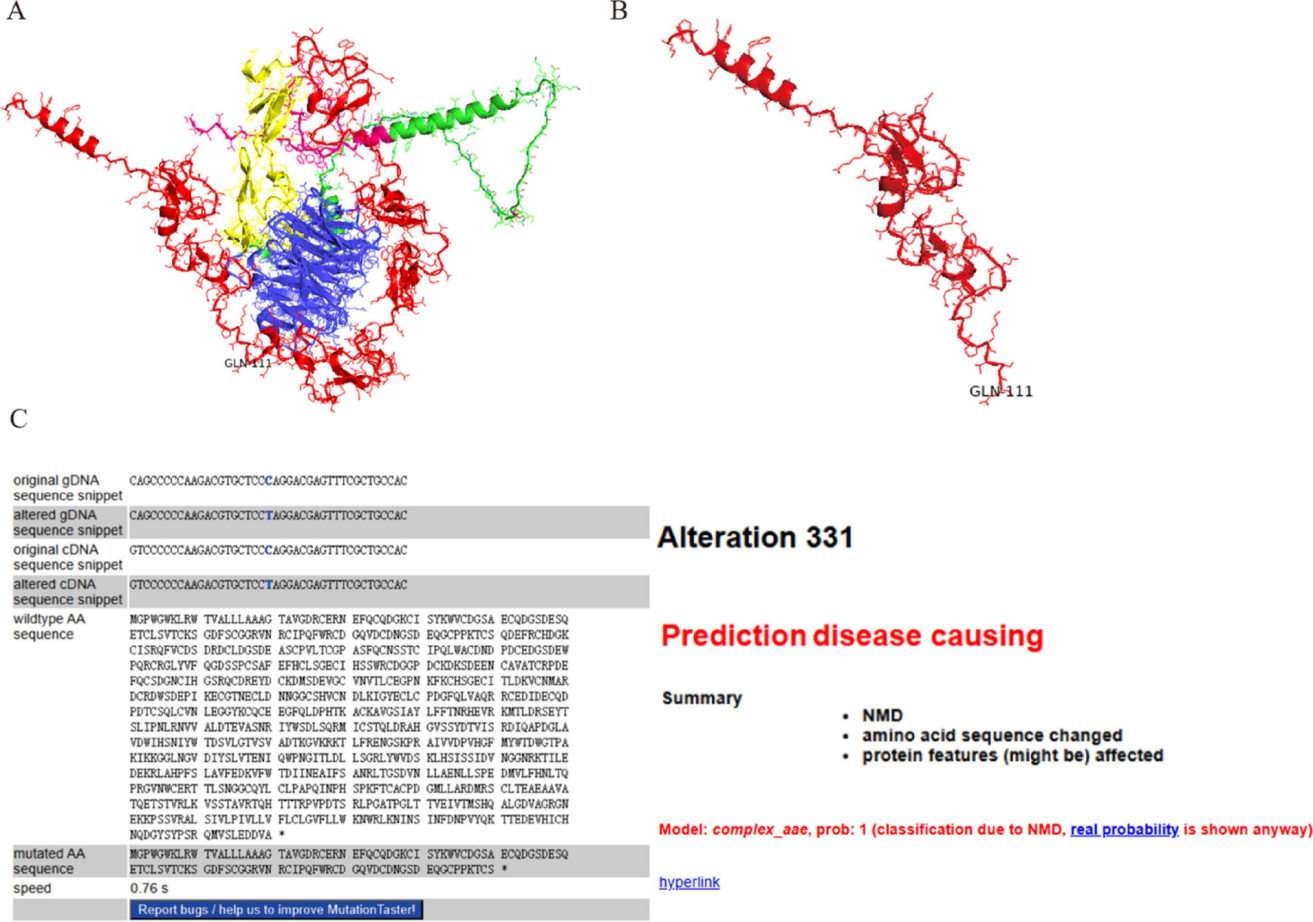
Predicted structural and functional consequences of the LDLR Protein of *LDLR* c.331C>T mutation. **(A)** Schematic representation of the normal LDLR protein conformation. **(B)** Predicted protein conformation resulting from the *LDLR* c.331C>T mutation. **(C)** Pathogenicity assessment of the *LDLR* c.331C>T mutation using MutationTaster.

The *LDLR* c.331C>T nonsense variant is located in exon 4 of the *LDLR* gene, within one of the seven repeats of the ligand-binding domain—a functionally critical region. Nonsense mutations in this region are highly likely to result in a severe disease phenotype. This domain is essential for the binding of LDLR to ApoB100, the major structural component of LDL and other lipoproteins. Disruption of this region is expected to substantially impair the ligand-binding capacity of LDLR (14). Given that this *de novo* mutation is a premature termination codon situated in an early exon (Figure 3), we predict that it leads to the production of a truncated protein lacking most functional domains, including part of the ligand-binding domain, the EGF-like domain, and the transmembrane domain. Consequently, key LDLR functions—such as ligand binding, internalization, and LDL uptake—are likely to be severely compromised. Furthermore, the mutant mRNA is highly susceptible to degradation via the nonsense-mediated mRNA decay (NMD) pathway (21), which would reduce mRNA levels from the mutant allele and further diminish *LDLR* expression.

### 1.3 Functional validation of *LDLR* c.331C>T mutation in Huh-7 cells

**Figure 4:**
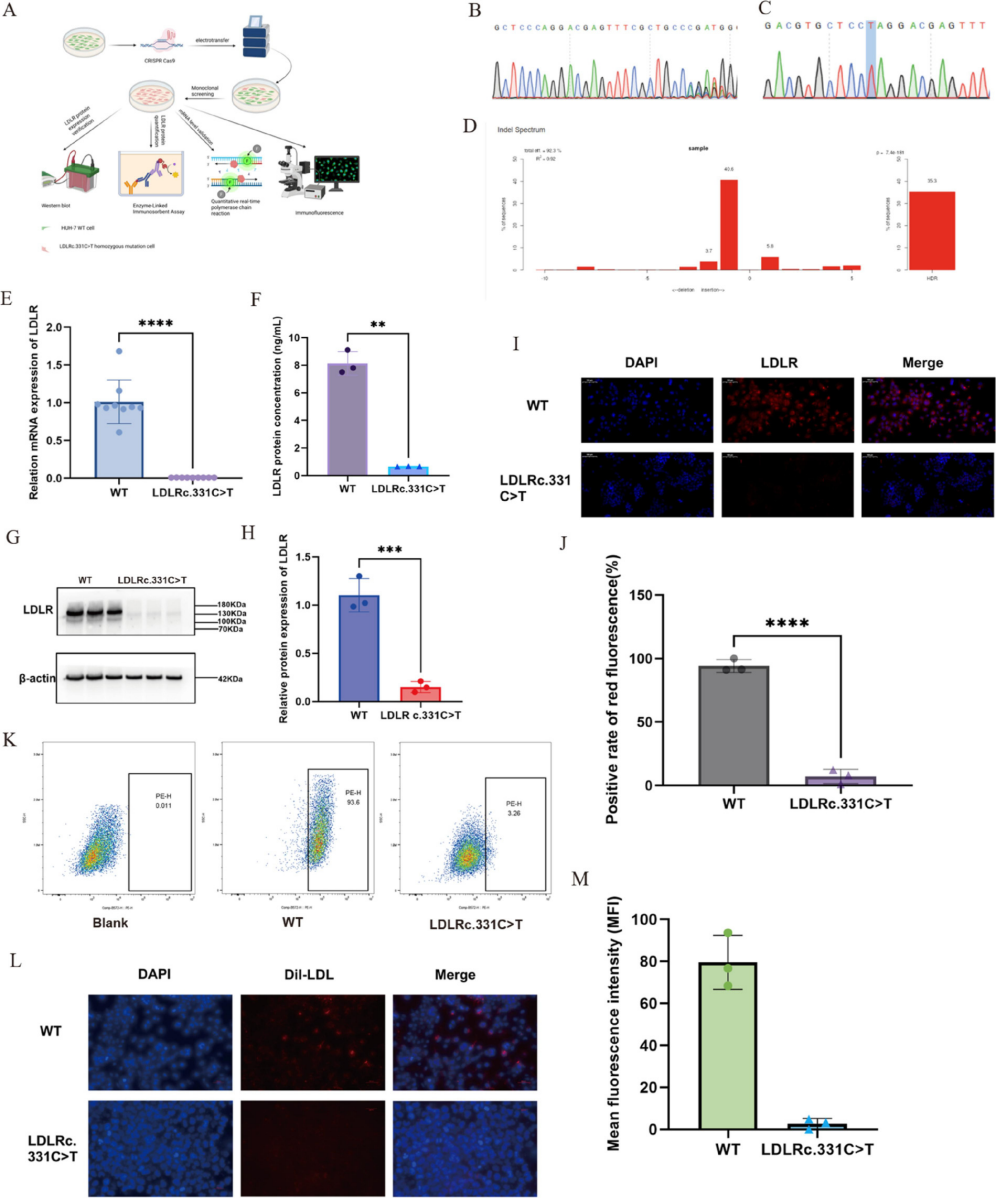
Construction and functional validation of *LDLR* c.331C>T cellular models. **(A)** Schematic diagram of the experimental workflow for functional validation. **(B)** Sanger sequencing chromatogram of HUH-7 cells following CRISPR-Cas9-mediated editing. **(C)** Sequencing chromatogram of the *LDLR* c.331C>T homozygous mutant clone isolated by limiting dilution. **(D)** HDR efficiency of CRISPR-Cas9 editing analyzed by TIDER. **(E)** Quantitative reverse transcription PCR (qRT-PCR) analysis of *LDLR* mRNA expression. **(F)** LDLR protein expression measured by ELISA in wild-type and *LDLR* c.331C>T mutant HUH-7 cells. **(G)** Western blot analysis of LDLR expression in wild-type and mutant cells. **(H)** Quantitative results from three independent replicates of western blot experiments. **(I)** Representative image of Immunofluorescence staining of LDLR (red) with DAPI nuclear counterstain (blue) in wild-type and mutant cells. **(J)** Comparison of positive rates in immunofluorescence assays. **(K)** Flow cytometric analysis of Dil-LDL fluorescence intensity. **(L)** Representative images of Dil-LDL fluorescence in cholesterol uptake assays observed under an inverted fluorescence microscope. **(M)** Quantitative analysis of DiI-LDL uptake by flow cytometry.

Using CRISPR-Cas9 technology, the *LDLR* c.331C>T point mutation was introduced into HUH-7 cells (Figure 4B). Following selection via limiting dilution, a homozygous *LDLR* c.331C>T mutant cell line was established (Figure 4C). Then, RT-Quantitative PCR analysis revealed significantly reduced mRNA expression of *LDLR* in the mutant cells compared with wild-type controls (Figure 4E). Western blotting results showed that wild-type HUH-7 cells displayed a prominent mature LDLR protein band at approximately 160 kDa, corresponding to fully glycosylated LDLR, along with a faint immature band near 120 kDa. In contrast, only a faint band at approximately 120 kDa was detected in the mutant cells. However, this band was not consistently observed across three independent Western blot experiments, and Sanger sequencing confirmed that the mutant cell line is 100% homozygous, ruling out the possibility of residual wild-type cell contamination. Combined with the qPCR results, we consider this faint band to be most likely non-specific, indicating that LDLR protein is virtually absent in the mutant cell line (Figure 4G). These results indicate that the expression of LDLR protein in the *LDLR* c.331C>T mutant cell line is significantly reduced.

Results of immunofluorescence staining demonstrated strong LDLR-associated fluorescence signal at the plasma membrane of wild-type cells using an anti-LDLR primary antibody followed by a red-fluorescent secondary antibody, indicating normal LDLR expression and localization. Quantitative analysis of at least 100 cells per condition revealed that the positive fluorescence rate in wild-type cells was 94.15%, whereas mutant cells showed a markedly lower positive rate of only 7.16%. In contrast, mutant cells exhibited markedly weaker fluorescence, reflecting impaired LDLR expression and cellular localization (Figure 4I, J). Collectively, these functional assays demonstrate that, compared with wild-type cells, the homozygous mutant cell line fails to efficiently express *LDLR* mRNA and protein. The absence of detectable LDLR signal at the cell membrane of mutant cells aligns with the predicted functional impact of the mutation.

The function of LDLR was evaluated by DiI-LDL uptake assay. Fluorescence microscopy observation (Figure 4L) showed abundant punctate red fluorescence signals on the cell membrane and in the cytoplasm of wild-type HUH-7 cells, indicating efficient LDLR-mediated binding and internalization of DiI-LDL. In contrast, almost no fluorescence signal was detectable in the *LDLR* c.331C>T homozygous mutant cell line (Figure 4L). Flow cytometric analysis (Figures 4K,M) further confirmed that the mean fluorescence intensity of the mutant cell line was significantly reduced. These results demonstrate that the c.331C>T nonsense mutation leads to severe impairment of LDLR-mediated LDL binding and uptake function.

### 1.4 Efficient and precise gene correction at the *LDLR* c.331C>T site

In this study, a PE-based gene correction system was designed for the *LDLR* c.331C>T site, consisting of four PE mRNAs (PE-DRNaseH, PEmax, PE6d and PE6c), three candidate epegRNAs (epegRNA1, epegRNA2 and epegRNA3) and a nickRNA sequence base on the target sequences. The correction efficiencies of the four PEmRNAs and three epegRNAs were systematically evaluated. The initial epegRNA, designated epeg1, was tested in combination with four PE enzymes previously shown to be effective in the study, along with the nickRNA, in the mutant cell line. Comparative analysis revealed that PE6c with epegRNA1 exhibited the highest correction efficiency.

**Figure 5:**
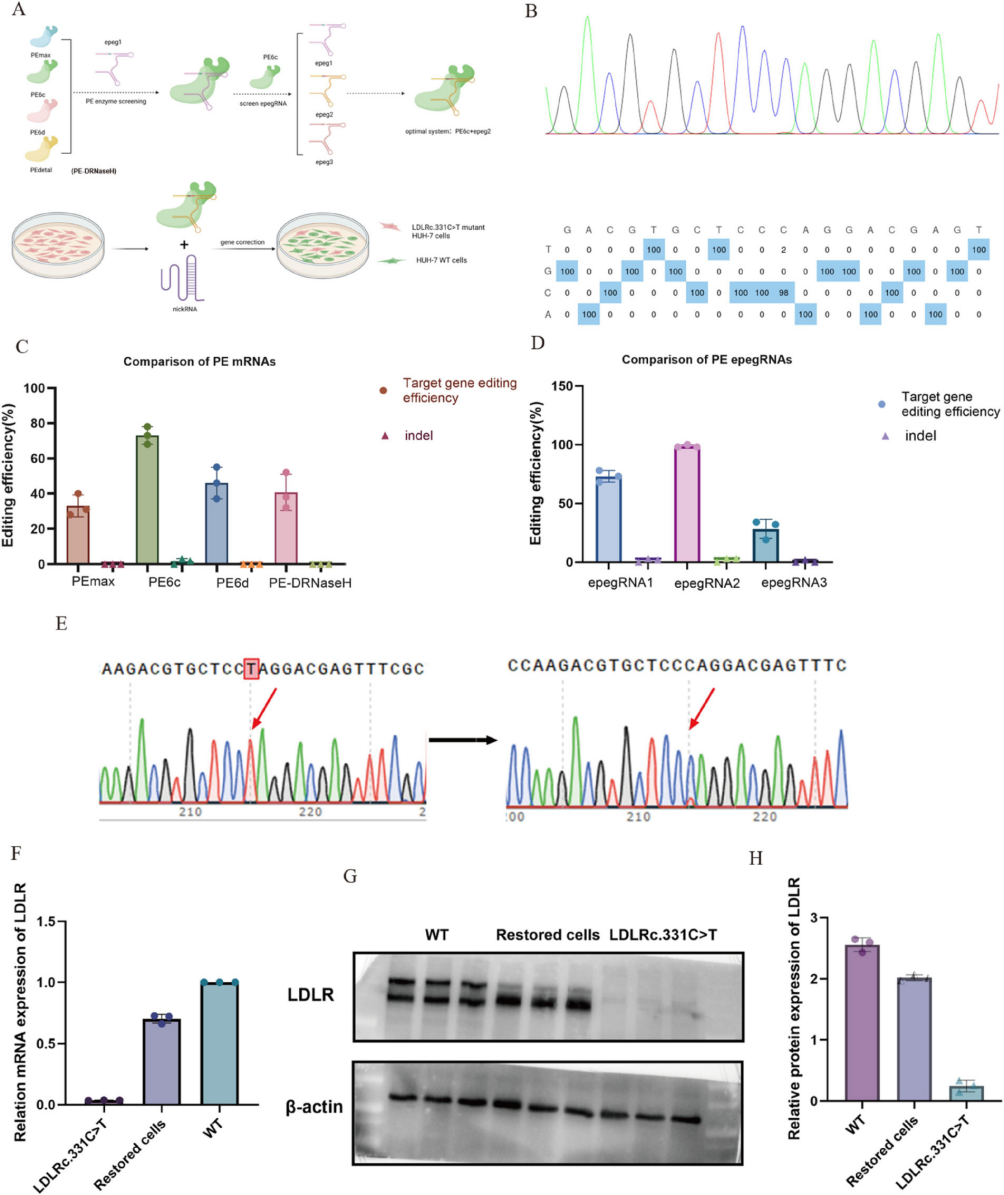
Screening and gene correction of PE system. **(A)** Schematic diagram of the screening and gene correction process for the PE system. **(B)** EdiR analysis of PE-adjusted *LDLR* point mutation peak graph. **(C)** Comparison of editing efficiency between PE enzymes. **(D)** Comparison of editing efficiency between epegRNAs. **(E)** Sanger sequencing chromatogram of the gene-corrected cell line. **(F)** LDLR mRNA expression levels in wild-type, homozygous mutant, and gene-corrected cells detected by qPCR. **(G)** LDLR protein expression in wild-type, homozygous mutant, and gene-corrected cells detected by Western Blot. **(H)** Densitometric quantification of LDLR protein levels normalized to *β*-actin.

Subsequently, two additional epegRNAs were designed and tested with PE6c for gene correction. The combination of PEc and epeg2 demonstrated the highest efficiency. Following electroporation into *LDLR* c.331C>T mutant Huh7 cells, Sanger sequencing results confirmed an approximately 98% high-fidelity correction at the target site. All experiments were performed in three independent replicates (Figure 5B).

To evaluate the therapeutic efficacy of the Prime Editing correction system, functional validation was performed on the corrected cell line. qPCR analysis (Figure 5F) demonstrated that the severely reduced LDLR mRNA levels in mutant cells were significantly restored following gene correction. Western Blot and densitometric analysis (Figures 5G,H) further confirmed the marked restoration of LDLR protein expression following gene correction. These results demonstrate that the Prime Editing correction system can rescue the functional defects caused by the LDLR c.331C>T mutation.

## 2 Material and methods

### 2.1 Bioinformatic structural prediction and pathogenicity analysis of the *de novo LDLR* mutation

The canonical amino acid sequence (UniProt ID: P01130; 860 aa) was retrieved from the UniProt database in FASTA format, aiming to obtain a complete three-dimensional (3D) model of the human wild-type LDLR protein. The 3D structure of wild-type LDLR was predicted using homology modeling via the SWISS-MODEL online server, with the canonical isoform as the template. The automatically generated model was optimized, saved in PDB format, and visualized using PyMOL.

A truncated mutant model was manually constructed in PyMOL by removing all residues downstream of position 111 for simulating the structural consequences of the identified *de novo* mutation (*LDLR* c.331C>T, p.Gln111Ter, heterozygous), corresponding to the predicted premature termination induced by the nonsense mutation. Comparative analysis of the folding, conformation, and surface features between the wild-type and mutant models provided an initial assessment of the potential structural alterations resulting from the variant.

The potential pathogenicity of the novel mutation *LDLR* c.331C>T (p.Gln111Ter, heterozygous) was assessed using the online prediction tool MutationTaster 2021. Input parameters included the gene name, the corresponding Ensembl transcript ID (ENST00000558518), the nucleotide sequence flanking the mutation site, and the specific variant details. The resulting predictions were recorded and further analyzed.

### 2.2 Construction of the CRISPR-Cas9 gene editing system

Design and synthesis of sgRNA: The genomic DNA sequence of *LDLR* (Gene ID: 3949) was retrieved from the NCBI database. Potential NGG protospacer adjacent motif (PAM) sites were identified within the target region, and the 20 bp sequence immediately upstream of each NGG site was selected as a candidate guide sequence. Candidate sgRNAs were generated using the sgRNA Designer tool (Broad Institute). Based on off-target scoring and on-target efficiency predictions, the optimal sgRNA was selected. The chosen 20 nt guide sequence (the spacer sequences are listed in Supplementary Table S1), together with the sgRNA scaffold (tracrRNA portion), the whole sequences of sgRNA were synthesized by GeneArt Precision gRNA Synthesis kit (A29377, Invitrogen™, ThermoFisher).

Design and synthesis of single strand donor DNA: The genomic template sequence containing the Cas9 cleavage site was retrieved and aligned with the HUH7 cell line reference. Flanking regions of 50nt length upstream and downstream of the cut site were selected for homology-directed repair. Donor DNA was designed in the following structure: left homology arm + *LDLR* mutation sequence + right homology arm, followed by codon optimization. The final single-strand oligodeoxynucleotide (ssODN) donor was commercially synthesized by Tsingke Biotechnology Co., Ltd. through custom oligonucleotide synthesis.

### 2.3 Cell culture

Huh-7 cells were maintained in DMEM supplemented with 10% fetal bovine serum (FBS; Gibco, Thermo Fisher Scientific, cat. no. 10099141C) and 1% penicillin-streptomycin (ViveCell, cat. no. AT052-100) at 37 °C in a humidified atmosphere containing 5% CO₂. When cells reached 80%–90% confluence, they were dissociated with 0.25% trypsin-EDTA (ViveCell, cat. no. AT010-100) for 3–5 min at 37 °C. After digestion, cells were washed once with DPBS, collected by centrifugation at 300×g for 5 min, and counted. Finally, cells were resuspended at a density of 1.0 × 10^5^ cells per well for subsequent experiments.

It is worth noting that prior to the functional assays, all cells used in this study were cultured in serum-free medium for 12 h to exclude the potential interference of lipoproteins on the experimental results.

### 2.4 Assembly of ribonucleoprotein (RNP) complexes and delivery via nucleofection

For each electroporation reaction, a 20 μL working solution was prepared using Solution P3 and the corresponding Supplement from the Lonza P3 Primary Cell 4D-Nucleofector™ X Kit (Lonza, cat. no. V4XP-3024). To form the ribonucleoprotein (RNP) complex, 1 μg of Cas9 protein (TrueCut™ Cas9 Protein v2, Invitrogen, cat. no. A36498) was incubated with 60 pmol of sgRNA at 37 °C for 15 min, followed by the addition of 100 pmol of single-stranded oligodeoxynucleotide (ssODN) donor. This mixture was then combined with the prepared working solution. The cell pellet was fully resuspended in the solution containing the RNP complex and transferred into a Nucleocuvette™. Electroporation was carried out using the 4D-Nucleofector™ X Unit with the predefined program CM-130. Immediately after pulsing, 80 μL of pre-warmed complete DMEM medium was added directly into the cuvette, and the cells were allowed to recover for 10 min at room temperature. Finally, the cell suspension was gently transferred to a 24-well plate and brought to a final culture volume of 500 μL with fresh complete medium. Cells were subsequently cultured under standard conditions for 48 h before further analysis.

### 2.5 Detection of gene editing efficiency

Collecting transfected cells and extracting genomic DNA. Huh7 cells harvested 48 h post-electroporation were collected by centrifugation. Genomic DNA was extracted using the Blood/Cell/Tissue Genomic DNA Extraction Kit (Tiangen Biotech, cat. no. DP304-03) according to the manufacturer's protocol. PCR amplification and sequencing. The genomic region encompassing the *LDLR* c.331 site was amplified by PCR using gene-specific primers (primer sequences are listed in Supplementary Table S1). The resulting PCR products were purified and subjected to Sanger sequencing. Editing efficiency analysis.

The sequencing chromatograms were analyzed using the TIDE (Tracking of Indels by Decomposition) or EditR software to quantify genome editing efficiency (indel frequency). All experiments were performed with at least three independent biological replicates.

### 2.6 Screening and identification of monoclonal *LDLR* c.331C>T homozygous mutant cell lines

Following confirmation of editing efficiency in the bulk cell population by sequencing, transfected cells were trypsinized and counted. Serial dilution was performed, and the cell suspension was seeded into a 96-well plate (Corning Costar, cat. no. 3599) at an approximate density of one cell per well to ensure that most wells contained single cells. Wells were monitored regularly, and those containing colonies derived from a single cell were marked. Once a monoclonal colony reached approximately 70% confluence in the well, the cells were trypsinized and transferred to a 48-well plate for further expansion. A portion of the expanded cells was collected for genomic DNA extraction. The genotype of each clone was analyzed using the procedures outlined in Method Section 5, which involved PCR amplification of the target locus followed by Sanger sequencing and analysis with TIDE/EditR. This screening and expansion process was repeated until monoclonal cell lines homozygous for the *LDLR* c.331C>T mutation were successfully isolated and validated.

### 2.7 Functional characterization of *LDLR* c.331C>T mutant cell lines

#### 2.7.1 Quantitative real-time polymerase chain reaction (qPCR)

Specific primers flanking the *LDLR* c.331 locus were designed (primer sequences are listed in Supplementary Table S1). Total cells' RNA was extracted from collected cells using the Eastep® Super Total RNA Extraction Kit (Promega, cat. no. LS1040). RNA concentration was measured with a NanoDrop 2000C spectrophotometer, and 1 µg of total RNA was reverse transcribed into cDNA using FastSenti Fast One-Step gDNA Removal and cDNA Synthesis SuperMix (TIANGEN, cat. no. KR138) following the manufacturer's protocol. Quantitative PCR (qPCR) was performed on a real-time PCR system with TB Green Premix Ex Taq II (Tli RNaseH Plus) (Takara Bio, cat. no. RR820) under the following cycling conditions: initial denaturation at 95  °C for 30 s, followed by 40 cycles of 95  °C for 5 s and 60  °C for 30 s. β-actin served as the endogenous reference gene (primer sequences listed in Supplementary Table S1). The relative expression level of *LDLR* mRNA was calculated using the 2−ΔΔCt method. All assays were performed in three independent biological replicates.

### 2.8 Western blot analysis

1. Protein extraction and quantification. Total protein was extracted from both wild-type and *LDLR* c.331C>T homozygous mutant Huh-7 cells using RIPA Lysis Buffer (Strong) (Proteintech, cat. no. PR20035) supplemented with Common Protease Inhibitor Cocktail (Proteintech, cat. no. PR20032). Protein concentration was determined using a BCA Protein Assay Kit (Proteintech, cat. no. PK10026). Protein samples (20 µg per lane) were mixed with 5×SDS-PAGE Protein Loading Buffer (Servicebio, cat. no. G2075), denatured at 98 °C for 10 min, and separated by electrophoresis on a 10% SDS-polyacrylamide gel.
2. Electrophoresis and membrane transfer. After separation, proteins were transferred onto a PVDF membrane (Immobilon-PSQ, Millipore, cat. no. ISEQ00010) using a wet transfer system. The membrane was blocked with 5% bovine serum albumin (BSA; Biofroxx, cat. no. 4240GR100) in TBST (Servicebio, cat. no. G0004) for 1 h at room temperature.
3. Antibody incubation. The membrane was incubated overnight at 4 °C with the primary antibody rabbit anti-LDLR Recombinant Monoclonal Antibody (Proteintech, cat. no. 82724-1-RR) diluted 1:6000 in blocking buffer. After three 7-min washes with TBST, the membrane was incubated for 1 h at room temperature with horseradish peroxidase (HRP)-conjugated goat anti-rabbit IgG secondary antibody (Signalway Antibody, cat. no. L3012) diluted 1:6000.
4. Chemiluminescence detection and normalization. Following another series of TBST washes, immunoreactive bands were visualized using an Enhanced Chemiluminescence (ECL) Detection Kit (Proteintech, cat. no. PK10001) and imaged with a Tanon chemiluminescence imaging system. To verify equal protein loading, the membrane was reprobed with rabbit anti-β-Actin (13E5) Monoclonal Antibody (Cell Signaling Technology, cat. no. 4970) as an internal control. Band intensities were quantified using ImageJ software (National Institutes of Health, Bethesda, MD, USA). The relative expression of LDLR protein was normalized to the corresponding β-Actin signal. All experiments were performed in three independent biological replicates.

### 2.9 Enzyme-linked immunosorbent sssay for LDLR protein quantification

Quantification of LDLR protein by ELISA. The concentration of LDLR protein in cell lysates was quantitatively measured using a Human LDLR (Low Density Lipoprotein Receptor) ELISA Kit (Elabscience, cat. no. E-EL-H1211) according to the manufacturer's instructions. Approximately 1 × 10⁶ cells were lysed in 200 μL DPBS containing protease inhibitor via three freeze-thaw cycles. After centrifugation at 12,000 × g for 15 min at 4 °C, supernatants were collected. To ensure measurements fell within the linear detection range, lysates from wild-type cells were diluted two-fold based on preliminary optimization. Following the manufacturer's protocol, samples and standards were incubated in pre-coated wells for 120 min at room temperature, followed by sequential incubation with biotinylated detection antibody and enzyme conjugate. TMB substrate was added for color development, and the reaction was stopped before measuring absorbance at 450 nm. A standard curve was generated from known concentrations, and LDLR levels in samples were calculated accordingly. All samples were analyzed in triplicate.

### 2.10 Immunofluorescence dtaining and vonfocal imaging

Wild-type and *LDLR* c.331C>T homozygous mutant Huh-7 cells were plated on confocal-compatible dishes (Biosharp, cat. no. BS-20-GJM) at a density of 1 × 10^5^ cells per dish and allowed to adhere for 24 h. Subsequently, cells were rinsed three times with phosphate-buffered saline (PBS) and fixed using ice-cold methanol (pre-chilled to −20  °C) for 15 min at ambient temperature. Following three additional PBS washes, nonspecific binding sites were blocked by incubating the cells in PBS supplemented with 5% bovine serum albumin (BSA) for 1 h at room temperature. Cells were then incubated with a rabbit-derived monoclonal anti-LDLR primary antibody (Proteintech, cat. no. 82724-1-RR)—identical to that employed in Western blot analyses—diluted 1:200 in blocking buffer, and left overnight at 4 °C. After extensive washing with PBS, samples were exposed for 1 h at room temperature in the dark to a fluorophore-conjugated secondary antibody [ABflo® 594-labeled goat anti-rabbit IgG (H + L), ABclonal, cat. no. AS039] at a dilution of 1:200. Following a final wash step, cell nuclei were visualized by counterstaining with 4′,6-diamidino-2-phenylindole (DAPI; ECOTOP, cat. no. ES-8245) for 10 min. Imaging was performed using a confocal laser scanning microscope. To evaluate LDLR protein expression qualitatively, the mean fluorescence intensity was compared between the two experimental groups.

### 2.11 Cholesterol uptake experiment

To evaluate the ligand-binding and endocytic function of the LDLR protein, cellular uptake assays were performed using DiI-labeled low-density lipoprotein (DiI-LDL). Qualitative analysis was conducted by fluorescence microscopy, and quantitative assessment was performed by flow cytometry.

Wild-type HUH-7 cells and LDLRc.331C>T homozygous mutant cells were seeded into 12-well plates at a density of 2 × 10^5^ cells per well. When cell confluence reached 70%–80%, the culture medium was replaced with serum-free DMEM, and cells were starved for 12 h in a 37 °C, 5% CO_2_ incubator to eliminate potential interference from serum lipoproteins. Following starvation, cells were incubated with serum-free DMEM containing 20 μg/mL DiI-LDL for 4 h in a 37 °C, 5% CO_2_ incubator under light-protected conditions. After incubation, the DiI-LDL-containing medium was aspirated, and cells were rapidly washed three times with ice-cold PBS (5 min each wash). Cells were then fixed with 4% paraformaldehyde for 15 min at room temperature, followed by DAPI nuclear staining for 10 min. After mounting with an anti-fade mounting medium containing a fluorescence quencher, images were acquired under a fluorescence microscope.

For flow cytometry analysis, cells in 12-well plates were digested with EDTA-free trypsin, centrifuged at 300 × g for 5 min, and the supernatant was discarded. Cells were resuspended in PBS containing 1% FBS and kept on ice protected from light.

Flow cytometric analysis was performed with an excitation wavelength of 549 nm and an emission detection channel of 565 nm (corresponding to the PE channel). The mean fluorescence intensity (MFI) was analyzed using FlowJo software and used as a quantitative indicator of LDL uptake capacity.

### 2.12 Statistical analysis

All quantitative data were derived from a minimum of three independent biological replicates and are expressed as mean ± standard deviation (SD). Statistical analyses were carried out using GraphPad Prism 9.0. Group comparisons between two conditions were evaluated with an unpaired two-tailed Student's *t*-test. For comparisons involving more than two groups, one-way analysis of variance (ANOVA) was applied, followed by Tukey's *post-hoc* test for multiple comparisons. Statistical significance was defined as *P* < 0.05.

### 2.13. Preparation of PE system components for mutation correction

#### 2.13.1 Preparation of PE protein mRNA template and *in vitro* transcription

The plasmid encoding PE6c (Addgene, plasmid #207853) was used as the DNA template. Linearized DNA template was generated via polymerase chain reaction (PCR). The resulting PCR product was purified using a QIAcuity Nanoplate 26k 24-well system (QIAGEN, cat. no. 250001). Purified DNA was quantified with a NanoDrop 2000C spectrophotometer, and its correct fragment size was confirmed by agarosegel electrophoresis before proceeding to *in vitro* transcription. *In vitro* transcription was performed using the HiScribe® T7 High Yield RNA Synthesis Kit (New England Biolabs, cat. no. E2040L) according to the manufacturer's protocol. Following transcription, PE protein mRNA was purified by lithium chloride precipitation and stored at −80 °C until use.

#### 2.13.2 Synthesis of nickRNA

A specific nickRNA primer targeting the *LDLR* c.331 site was designed (primer sequence provided in Supplementary Table S1). The corresponding oligo fragment was synthesized by Tsingke Biotechnology Co., Ltd. Using this oligo as the template, nickRNA was synthesized via *in vitro* transcription following the manufacturer's instructions of the GeneArt™ Precision gRNA Synthesis Kit (Invitrogen, cat. no. A29377).

#### 2.13.3 Design and synthesis of epegRNA

epegRNAs for correcting the *LDLR* c.331C>T mutation were designed according to the sequences provided in Supplementary Table S1. Corresponding oligonucleotides encoding the epegRNAs were commercially synthesized by GenScript Biotech. Both the 5′ and 3′ ends of each oligonucleotide were chemically modified at three terminal nucleoside (modified sequences are listed in Supplementary Table S1).

### 2.14 Reversion mutation electroporation and sequencing

The procedures for electroporation and sequencing to introduce and validate the reversion mutation were the same as described in the preceding sections “Detection of Gene Editing Efficiency”.

## 3 Discussion

The study reported a case of homozygous familial hypercholesterolemia (HoFH) presenting with classic clinical features including hypercholesterolemia, cutaneous xanthomas, and premature atherosclerosis. Genetic testing identified compound heterozygous mutations in the *LDLR* gene, consisting of a known frameshift mutation (LDLR c.1693–1696delGGCA) inherited from the mother and a *de novo* nonsense mutation (*LDLR* c.331C>T, p.Gln111Ter). Phenotypic analysis within the family revealed that her mother, a heterozygous carrier of the frameshift mutation alone, exhibited mild clinical manifestations that were controllable with medication, whereas the proband carrying both mutations developed severe symptoms from early childhood, suggesting that the *de novo* nonsense mutation likely exerts a profound impact on LDLR function.

To support the genetic interpretation and ACMG-based variant classification, we queried the ClinVar database. This variant is registered in ClinVar under Variation ID 1372829 (Accession: VCV001372829.5) and has been submitted by three institutions, including Labcorp Genetics, all consistently classifying it as pathogenic. The submitters' comments indicate that this nonsense variant is predicted to result in premature protein truncation or nonsense-mediated mRNA decay leading to loss of function, and that it has no frequency record in gnomAD. Of note, this variant has not been previously reported in patients with LDLR-related disorders in the published literature.

Bioinformatic predictions indicated that *LDLR* c.331C>T (p.Gln111Ter) is expected to introduce a premature termination codon after residue 111, resulting in a truncated protein that may disrupt key functional domains and trigger nonsense-mediated mRNA decay (NMD), thereby further exacerbating functional loss. This is completely consistent with the information in ClinVar. Through *in vitro* functional assays—including Western blotting, ELISA, qPCR, immunofluorescence, and cholesterol uptake assay—we confirmed that this mutation leads to absence of LDLR protein expression, abnormal subcellular localization, and impaired cholesterol uptake function.

Pathogenic mutations in *LDLR* can disrupt any stage of the LDLR cycle—synthesis, trafficking, ligand binding, internalization, or recycling—leading to impaired LDL-C clearance (15). The mutation reported in this study is located in exon 4, corresponding to the LA3 module of the ligand-binding domain. Previous studies have shown that the LA3–LA5 and LA7 modules are highly sensitive to mutations and directly participate in ApoB100 binding and LDL uptake (14, 22, 23), whereas alterations in LA1, LA2, and LA6 exert relatively minor effects (24). Therefore, the nonsense mutation in LA3 is predicted to result in the loss of most downstream structures, including critical binding modules, severely compromising LDL binding, internalization, and recycling (16), that is consistent with the functional deficits observed in our study.

The prevalence of FH has been increasing in recent years, yet diagnosis remains challenging (25). Many patients are only identified after developing cardiovascular complications (26), underscoring the urgent need to improve early detection and systematic management (27, 28). The identification and functional validation of this case further enrich the spectrum of pathogenic *LDLR* mutations and provide a basis for refining early screening, diagnosis, and management strategies for FH.

Despite advances in the diagnosis and mechanistic understanding of FH, treatment—particularly for HoFH patients with biallelic mutations—remains challenging. Current lipid-lowering regimens, such as statins, PCSK9 inhibitors and siRNA medicine (20, 29, 30), can only partially control lipid levels, do not address the underlying genetic defect, and are associated with side effects and the burden of lifelong therapy. Surgical interventions such as liver transplantation are limited in clinical practice (29, 31). It should be noted that for patients with HoFH who are refractory to pharmacological therapies, LDL apheresis remains a standard-of-care intervention. This procedure physically removes LDL-C from the circulation by extracorporeal adsorption or filtration, providing rapid and significant reductions in LDL-C levels. However, LDL apheresis is invasive, requires biweekly to monthly hospital visits, is costly, and offers only temporary benefit without addressing the underlying genetic defect. In recent years, the development of gene-editing technologies has opened new avenues for potentially curative treatments for FH. For example, in 2025, the base-editing (BE) drug YOLT-101 targeting PCSK9 received FDA approval to enter clinical trials, marking the formal transition of *in vivo* gene therapy for FH into clinical translation. Previously, studies in *LDLR* E208X mouse models demonstrated that AAV-delivered CRISPR-Cas9-mediated gene correction partially restored LDLR expression and improved atherosclerotic phenotypes (32), providing preliminary evidence for *LDLR*-targeted gene therapy.

The patient in this study faces multiple challenges: insufficient response to available lipid-lowering drugs, high cardiovascular risk (33), and limited access to liver transplantation. There is thus an urgent need for novel therapeutic strategies. Advances in precise gene-editing tools that avoid DSBs (such as PE) and delivery systems (including LNPs and engineered virus-like particles) offer technical possibilities for *in situ* correction of pathogenic point mutations (34, 35). In this study, we achieved highly efficient correction (∼98%) of the *LDLR* c.331C>T mutation using PE technology in a cellular model, laying a foundational proof-of-principle for developing personalized gene therapy targeting this specific variant. The recent report in NEJM of a child with CPS1 deficiency who achieved near-curative outcomes through individualized BE therapy further supports the feasibility of tailored gene-editing strategies for rare mutations (36).

Nevertheless, the study has several limitations that represent key challenges for clinical translation. First, validation was performed only in the HUH-7 hepatoma cell line, which differs from primary hepatocytes in cholesterol metabolic regulation; future studies should use patient-derived iPSC-differentiated hepatocytes or humanized animal models. Second, efficient and targeted *in vivo* delivery remains a major bottleneck; the safety, liver tropism, and cargo capacity of delivery systems such as LNPs and AAVs require further optimization. Third, the potential off-target effects of gene editing cannot be ignored, necessitating comprehensive off-target detection of the editing system to minimize the risks associated with off-target editing, as well as long-term safety assessments in animal models. Genome-wide off-target editing risks of this PE system need to be systematically evaluated in primary cells. Furthermore, unintended germline editing may occur, and such editing is irreversible, with mutations being passed down through generations, raising significant ethical concerns. The long-term efficacy, stability, and potential oncogenic risks of gene editing must be monitored in long-term animal studies. Therefore, rigorous preclinical safety assessments are essential. Only after thorough long-term safety and ethical evaluations can this technology be truly applied in clinical practice. Fifth, the study focused on validating the pathogenicity of c.331C>T and did not functionally analyze the coexisting c.1693–1696delGGCA mutation; therefore, potential synergistic effects of the two mutations cannot be excluded and warrant further investigation. Sixthly, an additional limitation of this study concerns the strategy for selecting and validating homozygous mutant cell clones. From our limiting dilution screening, we identified three independent single clones carrying the LDLR c.331C>T homozygous mutation. However, we did not perform systematic functional characterization on each of these three clones separately. Because functional assays (including Western blot, immunofluorescence, ELISA, and LDL uptake assays) require large numbers of cells, we pooled two of the homozygous mutant cell lines for functional validation, rather than analyzing each independent clone individually. We acknowledge that, although Sanger sequencing confirmed the genotype of each clone with clean backgrounds at the editing site and no observed off-target editing, this is not sufficient to fully exclude the potential impact of clonal variability on functional phenotypes. This represents a methodological limitation of the present study. Future studies incorporating at least two to three independently derived homozygous clones for separate functional validation will be necessary to further confirm the reproducibility and reliability of our findings.

In summary, this study not only expands the spectrum of pathogenic *LDLR* mutations and deepens the understanding of how nonsense mutations contribute to severe FH phenotypes, but, more importantly, provides essential target validation and preliminary technical feasibility data for developing a mutation-specific gene-therapy strategy. Future research will focus on optimizing the editing system in animal models and patient-derived organoids, exploring safe and efficient delivery platforms, and conducting comprehensive safety and efficacy evaluations to advance clinical translation, ultimately offering new diagnostic and therapeutic hope for patients carrying such rare mutations.

## Data Availability

The original contributions presented in the study are included in the article/Supplementary Material, further inquiries can be directed to the corresponding author.
