# Supplementary material for: A de novo LDLR mutation in severe familial hypercholesterolemia: case report, functional characterization, and a personalized gene correction strategy exploration
Source: Front Cardiovasc Med. 2026 Jun 9;13:1814798. doi: 10.3389/fcvm.2026.1814798 (PMC13286746; doi:10.3389/fcvm.2026.1814798)
Supplement: Supplementary file 1 [file Table1.docx]

Supplementary Table S1：

| Sequence Name | Sequence | Use |
| --- | --- | --- |
| LDLR-331 F | TACACCTATTAGCGCACCAGT | PCR amplification and Sanger sequencing |
| LDLR-331 F | CATCCGAGCCATCTTCGCA | PCR amplification and Sanger sequencing |
| LDLR-qPCR-F | TGCTTGTCTGTCACCTGCAA | qPCR |
| LDLR-qPCR-R | GCAGCGAAACTCGTCCTGG | qPCR |
| LDLR-c.331-sgRNA | GTGGCAGCGAAACTCGTCCTGTTTTAGAGCTAGAAATAGCAAGTTAAAATAAGGCTAGTCCGTTATCAACTTGAAAAAGTGGCACCGAGTCGGTGC | CRISPR/Cas9 sgRNA |
| LDLR-ODN-100bp | TCTCGGCCCATCCATCCCTGCAGCCCCAGACGTGCTCCCAGGACGAGTTTCGCTGCCACGATGGGAAGTGCATCTCTCGGCAGTTCGTCTGTGACTCA | CRISPR/Cas9 sgRNA |
| β-actin-qPCR-F | CCTGGCACCCAGCACAAT | As the reference gene for the qPCR experiment |
| β-actin-qPCR-R | GGGCCGGACTCGTCATAC | As the reference gene for the qPCR experiment |
| LDLR-331-W-epegRNA1 | GTGGCAGCGAAACTCGTCCTGTTTCAGAGCTATGCTGGAAACAGCATAGCAAGTTGAAATAAGGCTAGTCCGTTATCAACTTGAAAAAGTGGCACCGAGTCGGTGCGTGCTCCcAGGACGAGTTTCGCTGCAGGGAACCCGCGGTTCTATCTAGTTACGCGTTAAACC | PE correction system |
| LDLR-331-W-epegRNA2： | GTGGCAGCGAAACTCGTCCTGTTTCAGAGCTATGCTGGAAACAGCATAGCAAGTTGAAATAAGGCTAGTCCGTTATCAACTTGAAAAAGTGGCACCGAGTCGGTGCGTGCTCCcAGGACGAGTTTCGCTGGAAGAAGGCGCGGTTCTATCTAGTTACGCGTTAAACCAACTAGAA | PE correction system |
| LDLR-331-W-epegRNA3 | GGCCCCCAAGACGTGCTCCTGTTTCAGAGCTATGCTGGAAACAGCATAGCAAGTTGAAATAAGGCTAGTCCGTTATCAACTTGAAAAAGTGGCACCGAGTCGGTGCCAGCGAAACTCGTCCTgGGAGCACGTCTTGGTAATAATGCGCGGTTCTATCTAGTTACGCGTTAAACCAACTAGAA | PE correction system |
| LDLR-c.331-nick RNA | GAGAGATGCACTTCCCATCGTTTTAGAGCTAGAAATAGCAAGTTAAAATAAGGCTAGTCCGTTATCAACTTGAAAAAGTGGCACCGAGTCGGTGC | PE correction system |
